# The Relevance of Hyperuricemia and Metabolic Syndrome and the Effect of Blood Lead Level on Uric Acid Concentration in Steelmaking Workers

**DOI:** 10.1186/2052-4374-25-27

**Published:** 2013-10-25

**Authors:** Deul Lee, Won-Jun Choi, Jae-Seok Oh, Min-Kee Yi, Sung-Woo Han, Jong-Wan Yun, Sang-Hwan Han

**Affiliations:** 1 Department of Occupational & Environmental Medicine, Gachon University Gil Medical Center, Incheon, Korea

**Keywords:** Lead, Uric acid, Metabolic syndrome

## Abstract

**Objectives:**

Uric acid concentration is known to increase the prevalence of metabolic syndrome by affecting its components, resulting in increased risk of cerebrovascular and cardiovascular diseases, and long-term lead exposure is known to affect this serum uric acid level. In this study, we aimed to examine the association between the causes of hyperuricemia and metabolic syndrome, and to determine whether an increased blood lead level affects hyperuricemia.

**Method:**

Anthropometric measurements, surveys, and blood tests were conducted between May and June 2012 in 759 men working in the steelmaking process at a domestic steel company. Workers were divided into 2 groups according to the presence or absence of hyperuricemia, and an analysis was performed to examine its association with metabolic syndrome. In addition, the workers were divided into 3 groups according to the blood lead level to analyze the association between blood lead and hyperuricemia.

**Results:**

The geometric mean (standard deviation) of the blood lead levels in the hyperuricemia group was significantly higher than that of the healthy group (3.8 [1.8] vs. 3.3 [1.8] μg/dL). The adjusted odds ratio for metabolic syndrome of the hyperuricemia group increased significantly to 1.787 (1.125–2.839) compared with the healthy group. In addition, the adjusted odds ratios for the occurrence of hyperuricemia in the tertile 2 (2.61–4.50 μg/dL) and tertile 3 groups (>4.50 μg/dL) according to blood lead level significantly increased to 1.763 (1.116–2.784) and 1.982 (1.254–3.132), respectively, compared with the tertile 1 group (< 2.61 μg/dL).

**Conclusion:**

Hyperuricemia is believed to function as an independent risk factor for metabolic syndrome, while lead seems to increase the serum uric acid level even at a considerably low blood level. Therefore, attention should be given to patients with hyperuricemia and metabolic syndrome who are prone to lead exposure, and a prospective study should be conducted to identify their causal relationship.

## Introduction

According to the 2011 Statistics Korea data on domestic causes of death, cerebrovascular and heart diseases ranked second and third, respectively, among the 3 leading causes of death in Korea as yet [1]. Moreover, according to a report, 278 of 731 occupational disease-related deaths in 2011 were due to cerebrovascular and cardiovascular diseases [2]. As such, the prevention of occupational cerebrovascular and cardiovascular diseases is gaining importance along with continuous efforts of many businesses and health institutions to develop practical preventive measures through the management of metabolic syndromes that involve various cerebrovascular and cardiovascular risk factors.

Various studies have identified metabolic syndromes as a major predictor of cerebrovascular and cardiovascular diseases [3], and study results indicated that the occurrence and progression of cerebrovascular and cardiovascular diseases can be prevented through strict management of metabolic syndrome [4].

Recently, continuous reports have emerged on the association between metabolic syndrome, cerebrovascular and cardiovascular diseases, and serum uric acid level, which is well known to be a causal factor for gout. Gradually accumulated evidence support that serum uric acid level has a significant correlation with each component of a metabolic syndrome, as it causes hypertension and insulin resistance and is involved in changes in blood triglycerides and cholesterol levels while being associated with obesity and complications of metabolic syndromes [5,6].

Existing studies conducted with the general population or workers reported their analysis results that hyperuricemia significantly correlated with blood lead concentrations [7]. Meanwhile, the results of other studies verified relatively high serum uric acid levels and high incidence rates of gout in people with long-term lead exposure [8,9]; as such, an integrated approach for the management of long-term lead exposure and hyperuricemia, as well as metabolic syndrome, is necessary from the perspective of occupational environment medicine.

Until the present, only a few domestic studies have analyzed the relationship between hyperuricemia and metabolic syndrome; in particular, it is difficult to find domestic and international case studies analyzing the relationship between occupational lead exposure, hyperuricemia, and metabolic syndrome. Therefore, this study aimed to examine the relationship between blood lead level and serum uric acid concentration, and the association between hyperuricemia and the occurrence of metabolic syndrome in male workers involved in steelmaking [10], which requires the most number of lead handling processes among metal industries.

## Materials and methods

### Subject

Health screenings were conducted for a domestic steelmaker between May 15 and June 5, 2012, in which 2,133 of total 2,157 target workers were screened. Among the target workers, most were male workers, accounting for 2,097 subjects (97.2%), 765 (36.5%) of whom were working in the steelmaking process. Among them, 759 male workers participated in the survey. Excluding the 6 male steelmaking workers who did not participate in the anthropometry or blood testing or who provided inadequate responses to the survey, a total of 759 workers were finally selected as research subjects.

### Anthropometric measurements and surveys

Height and weight were measured using an automatic body measurement device, and a tape measure was used to measure the waist circumference on the midpoint between the bottom part of the ribs and the upper part of the pelvic bones horizontal to the ground. Blood pressure was measured using an automatic blood pressure measuring system after more than 5 minutes of steady state; when the systolic and diastolic pressures were 140 and 90 mm Hg or higher, respectively, the lower value was selected after a repeated measurement taken after more than 10 minutes of rest. Body mass index (BMI) was calculated by dividing weight (kg) by the square of height (m).

Through a self-reported survey, medical history of hypertension, diabetes, and dyslipidemia, and the use of medication therapy, smoking and drinking habits, and exercise, were examined. Survey data on smoking and drinking habits (considering the different types of alcohol) were presented in pack-years and days/week, respectively. *Exercise* was defined as a minimum of 20 minutes of workout per day until feeling out of breath and was expressed in frequency (days/week).

### Blood and urine tests

After at least 8 hours of fasting, venous blood samples were collected from the subjects and fasting glucose, total cholesterol, triglyceride, high-density lipoprotein (HDL) cholesterol, aspartate aminotransferase (AST), alanine aminotransferase (ALT), γ-glutamic transpeptidase (γ-GTP), creatinine, blood lead, and serum uric acid concentrations were measured. The blood lead concentration analysis was performed using atomic absorption spectrophotometry (AAnalyst 600, PerkinElmer, Finland), and serum uric acid concentration was measured using Uricase EMST method (Hitachi 747 automatic analyzer, Hitachi, Tokyo, Japan). For low-density lipoprotein (LDL) cholesterol, the value calculated using the Friedewald formula, LDL (mg/dL) = TC (mg/dL) - HDL (mg/dL) - TG (mg/dL)/5, was used when the triglyceride level was lower than 400 mg/dL and the direct measurement method through the homogenous method was used if the triglyceride level exceeded 400 mg/dL.

### Definition of metabolic syndrome

By applying the criteria of the American Heart Association/National Heart Lung and Blood Institute published in 2005 [11], metabolic syndrome was defined when 3 or more of the following 5 risk factors were applicable: (1) waist circumference of 90 cm or greater, (2) blood triglyceride concentrations of 150 mg/dL or higher or being under medication therapy, (3) blood HDL level lower than 40 mg/dL or being under medication therapy, (4) systolic pressure of 130 mm Hg or higher or diastolic pressure of 85 mm Hg or higher or being under medication therapy, and (5) fasting glucose level of 100 mg/dL or higher or being under medication therapy.

### Statistical analysis

Statistical analysis was performed using SPSS (version 18.0). *Hyperuricemia* was defined as a serum uric acid concentration of higher than 7.0 mg/dL [12], and a *t* test was performed to determine whether a hyperuricemia-dependent difference existed in the anthropometric measurements, blood test results, and living habits. As blood lead concentration showed a distribution skewed to the left, geometric means and standard deviations were calculated after performing natural log transformation. To analyze the relationship between hyperuricemia and metabolic syndrome, we performed a logistic regression analysis. For variables other than hyperuricemia, a multivariate logistic regression analysis was performed by including them in the model as confounding variables after confirming multicollinearity. In addition, the relationship between blood lead concentration and hyperuricemia was analyzed. Blood lead concentration was set as an explanatory variable by classifying it in tertiles, and a multivariate logistic regression analysis was performed by including them in the model as confounding variables after confirming multicollinearity among other variables, excluding the components of metabolic syndrome.

## Results

### Hyperuricemia-dependent clinical characteristics of the subjects

The healthy (without hyperuricemia) group had 577 subjects, and the hyperuricemia group had 182 subjects. The mean (standard deviation) for serum uric acid concentration was 5.5 (0.9) mg/dL in the healthy group and 7.8 (0.7) mg/dL in the hyperuricemia group (Table 1). Compared with the healthy group, the mean age was significantly lower and the mean waist circumference and BMI were significantly higher in the hyperuricemia group. In the hyperuricemia group, fasting glucose and HDL-cholesterol levels were significantly lower, whereas triglycerides, AST, ALT, γ-GTP, and serum creatinine levels were significantly higher compared to those in the healthy group. Weekly drinking frequency was significantly higher but weekly exercise frequency was significantly lower in the hyperuricemia group than in the healthy group. The geometric mean (standard deviation) of the blood lead concentrations was significantly higher in the hyperuricemia group, at 3.8 (1.8) μg/dL, than in the healthy group, at 3.3(1.8) μg/dL.

**Table 1:** Hyperuricemia-dependent clinical characteristics of the subjects

**Variables**	**Normal (N = 577)**		**Hyperuricemia**^**† **^**(N = 182)**		**p-value**
	**Mean (SD)**		**Mean (SD)**		
Age (years)	42.3	(10.9)	38.1	(10.3)	0.000^*^
Waist circumference (cm)	83.5	(7.2)	85.5	(7.5)	0.001^*^
Body mass index (kg/m^2^)	24.1	(2.6)	24.9	(2.9)	0.001^*^
Systolic blood pressure (mmHg)	118.3	(12.1)	119.8	(11.5)	0.135
Diastolic blood pressure (mmHg)	76.2	(9.7)	77.4	(9.8)	0.142
Fasting blood glucose (mg/dL)	98.5	(21.4)	94.0	(11.5)	0.000^*^
Total cholesterol (mg/dL)	194.0	(34.6)	199.7	(34.9)	0.054
LDL-cholesterol (mg/dL)	110.0	(30.7)	110.6	(34.8)	0.829
HDL-cholesterol (mg/dL)	54.0	(11.9)	51.1	(10.9)	0.004^*^
Triglyceride (mg/dL)	157.0	(116.0)	209.0	(161.4)	0.000^*^
AST (IU/L)	24.5	(13.3)	28.7	(14.7)	0.001^*^
ALT (IU/L)	27.4	(25.8)	35.2	(27.3)	0.001^*^
γ-GTP (IU/L)	44.0	(46.7)	59.2	(60.8)	0.002^*^
Serum creatinine (mg/dL)	0.9	(0.1)	1.0	(0.1)	0.000^*^
Smoking (pack-year)	10.7	(11.9)	11.2	(11.6)	0.622
Drinking (days/week)	1.7	(1.9)	1.9	(1.3)	0.037^*^
Exercise (days/week)	1.8	(1.7)	1.5	(1.7)	0.029^*^
Blood lead (μg/dL)^‡^	3.3	(1.8)	3.8	(1.8)	0.003^*^

^*^: p < 0.05 by t-test.

^†^: Serum uric acid level ≥7 mg/dL.

^‡^: Geometric mean (Geometric standard deviation).

### Relationship between hyperuricemia and the occurrence of metabolic syndrome

Hyperuricemia was found to be significantly associated with the occurrence of metabolic syndrome through the univariate (model 1; Table 2) and multivariate logistic regression analyses, the latter being adjusted for age and blood lead concentration (model 2); additionally adjusted for smoking, drinking, and exercise habits (model 3); and for LDL-cholesterol, γ-GTP, and serum creatinine levels (model 4). In addition, age and γ-GTP level were found to be significantly associated with metabolic syndrome.

**Table 2:** Multiple logistic regression analysis demonstrating relationship between hyperuricemia and metabolic syndrome^* ^**in four models**

**Variables**	**Odds ratio (95% confidence interval)**			
	**Model 1**	**Model 2**	**Model 3**	**Model 4**
Hyperuricemia (≥7 mg/dL)				
No	1.000	1.000	1.000	1.000
Yes	1.533 (1.014 ~ 2.317)	2.016 (1.296 ~ 3.135)	1.939 (1.242 ~ 3.026)	1.787 (1.125 ~ 2.839)
Age (years)		1.057 (1.037 ~ 1.077)	1.055 (1.032 ~ 1.077)	1.049 (1.026 ~ 1.073)
log transformed blood lead (μg/dL)		0.855 (0.414 ~ 1.768)	0.822 (0.394 ~ 1.715)	0.789 (0.370 ~ 1.681)
Smoking (pack-year)			1.007 (0.991 ~ 1.024)	1.005 (0.988 ~ 1.022)
Drinking (days/week)			1.035 (0.906 ~ 1.183)	0.948 (0.820 ~ 1.097)
Exercise (days/week)			0.941 (0.837 ~ 1.057)	0.969 (0.861 ~ 1.091)
LDL-cholesterol (mg/dL)				1.002 (0.996 ~ 1.008)
γ-GTP (IU/L)				1.008 (1.005 ~ 1.012)
Serum creatinine (mg/dL)				0.433 (0.077 ~ 2.442)

^*^: Defined as the presence of at least three of the following components : waist circumference ≥90 cm, triglycerides ≥150 mg/dL or on drug treatment, HDL-cholesterol < 40 mg/dL or on drug treatment, SBP ≥130 or DBP ≥85 mmHg or on drug treatment, and fasting glucose ≥100 mg/dL or on drug treatment.

Model 1: not adjusted.

Model 2: adjusted by age, log transformed blood lead.

Model 3: adjusted for model 2 plus smoking, drinking, exercise.

Model 4: adjusted for model 3 plus LDL-cholesterol, γ-GTP, serum creatinine.

### Association between blood lead concentration and the occurrence of hyperuricemia

In the univariate logistic regression analysis (model 1), the odds ratio for hyperuricemia increased as the blood lead concentration increased in relation to 1 quartile (Table 3). Blood lead concentration was found to have a significant association with hyperuricemia in the multivariate logistic regression analysis models respectively adjusted for age, BMI, and γ-GTP level (model 2); additionally adjusted for smoking, drinking, and exercise habits (model 3); and adjusted for serum creatinine (model 4). In addition, a significant association was observed between hyperuricemia, and BMI, γ-GTP level, smoking habit, and serum creatinine level, among which serum creatinine level particularly showed a high odds ratio of 16.877, indicating a very high degree of association with hyperuricemia.

**Table 3:** Multiple logistic regression analysis demonstrating association between blood lead level and hyperuricemia^* ^**in four models**

**Variables**	**Odds ratio (95% confidence interval)**			
	**Model 1**	**Model 2**	**Model 3**	**Model 4**
Tertile group (blood lead, μg/dL)				
I (< 2.61)	1.000	1.000	1.000	1.000
II (2.61 ~ 4.50)	1.676 (1.091 ~ 2.576)	1.971 (1.258 ~ 3.087)	1.902 (1.211 ~ 2.989)	1.763 (1.116 ~ 2.784)
III (>4.50)	1.890 (1.236 ~ 2.889)	2.286 (1.460 ~ 3.579)	2.182 (1.389 ~ 3.430)	1.982 (1.254 ~ 3.132)
Age (years)		0.951 (0.934 ~ 0.967)	0.943 (0.924 ~ 0.962)	0.945 (0.925 ~ 0.965)
Body mass index (kg/m^2^)		1.098 (1.031 ~ 1.168)	1.099 (1.033 ~ 1.170)	1.096 (1.030 ~ 1.168)
γ-GTP (IU/L)		1.006 (1.003 ~ 1.010)	1.005 (1.002 ~ 1.009)	1.005 (1.002 ~ 1.009)
Smoking (pack-year)			1.017 (0.999 ~ 1.035)	1.020 (1.002 ~ 1.039)
Drinking (days/week)			1.076 (0.944 ~ 1.226)	1.085 (0.951 ~ 1.238)
Exercise (days/week)			0.950 (0.853 ~ 1.058)	0.938 (0.841 ~ 1.046)
Serum creatinine (mg/dL)				16.877 (3.388 ~ 84.073)

^*^: Serum uric acid level ≥7 mg/dL.

Model 1: not adjusted.

Model 2: adjusted by age, body mass index, γ-GTP.

Model 3: adjusted for model 2 plus smoking, drinking, exercise.

Model 4: adjusted for model 3 plus serum creatinine.

## Discussion

This study examined the association between hyperuricemia and metabolic syndrome, and the association between blood lead and serum uric acid concentrations. Hyperuricemia showed a significant association with metabolic syndrome even in the results adjusted for confounding variables, whereas lead was shown to be associated with hyperuricemia even at low blood concentrations.

According to existing studies [13,14], an increase in serum uric acid concentration is thought to partially contribute to an increase in blood pressure via the activation of the renin-angiotensin-aldosterone system by inducing renal vascular inflammation, preglomerular arteriolopathy, tubulointerstitial inflammation and fibrosis. Serum uric acid concentration also seems to interfere with blood vessel expansion by decreasing the response of blood vessels to acetylcholine and to increase blood pressure by causing endothelial dysfunction through interference in the excretion process of nitric oxide (NO) from vascular endothelial cells by expressing toxicity directly on the blood vessels [15,16]. In this regard, a study reported that when the activity of xanthine oxidase, which is involved in the uric acid generation process, was inhibited by administering allopurinol to patients, blood flow was improved along with vasodilation [17].

Whether serum uric acid level independently influences the increase in blood glucose level is controversial. While there are reports on increased insulin resistance due to certain serum uric acid levels increasing blood flow and causing vasodilation to interfere with the action of nitric oxide, which facilitates glucose absorption [18], other results suggest that hyperuricemia is caused by hyperinsulinemia due to insulin resistance acting on the renal tubules to facilitate the reabsorption of uric acid [19,20]. These results suggest an interaction between uric acid and insulin levels. However, the mean fasting glucose level in the hyperuricemia group was significantly lower than that in the healthy group in this study, contradicting the results of existing studies. Upon considering the results of recent related studies, a decreased fasting glucose level in patients with hyperuricemia is possibly due to the increase in uric acid excretion caused by the glomerular hyperfiltration when hyperglycemia is maintained [21,22]. However, additional research is necessary in the future to validate this hypothesis.

The effects of serum uric acid level on dyslipidemia have not yet been clearly identified; however, according to recent studies, the increase in uric acid concentration seems to reduce the function of lipid peroxidase and lipoprotein lipase, which are involved in lipolysis, while the process of decreased nitric oxide excretion from blood vessels is believed to affect the progression of dyslipidemia [23,24]. In this study, the total cholesterol and LDL-cholesterol levels in the hyperuricemia group were not statistically significant, but the mean levels were still higher compared to those in the healthy group. Meanwhile, triglyceride levels were significantly higher in the hyperuricemia group, with significantly lower HDL-cholesterol levels, than in the healthy group. In particular, the levels of the triglycerides related to metabolic syndrome showed the greatest difference in mean values when compared with the other parameters, which seems to be due to the decreased function of the tricarboxylic acid cycle with the increased oxidative stress in the mitochondria of liver cells as the uric acid synthesis increased due to purine metabolism, resulting in slower triglyceride metabolism and an increase in lipogenesis [25].

Regarding the association between obesity and serum uric acid concentration, it is known that obese individuals mostly consume meat, with high intake of purine, which results in an increase in uric acid concentration [26], and hyperuricemia is caused by the facilitation of uric acid reabsorption in the renal tubules due to high insulin resistance. These results point to obesity as the cause of hyperuricemia [27,28]. However, based on the various correlations identified so far, such as the close association between insulin resistance and nitric oxide synthesis dysfunction in vascular endothelial cells and the claim that uric acid interferes with the nitric oxide excretion process [18], the increase in the risk for fatty liver in proportion to the purine metabolism-induced uric acid synthesis triggered by the deterioration in mitochondria function in liver cells [25], and the contribution of the reduced mitochondria functioning to the fat accumulation in muscles and liver in a study with a non-obese elderly population [29], hyperuricemia itself seems to partially contribute to obesity. This finding warrants additional research in the future.

In this study, the hyperuricemia group showed a significantly higher mean blood lead concentration than that in the healthy group. The subjects’ geometric mean (standard deviation) blood lead concentration was approximately 3.4 (1.8) μg/dL, which was significantly lower than 25.0 μg/dL [30], the blood lead concentration designated by the Centers for Disease Control and Prevention (CDC) as affecting the physiological health of adults, and even below the recommended upper limit level of 5.0 μg/dL for children in 2012 [31]. However, in our univariate and multivariate regression analyses, the risk of hyperuricemia was shown to increase even at low blood lead concentrations between 2.61 and 4.50 μg/dL.

Uric acid is synthesized through the metabolism of purine, and humans are known to have a relatively high level of serum uric acid concentration compared with other mammals, as humans do not possess the enzyme for metabolizing uric acid [32]. Therefore, to maintain an appropriate level of serum uric acid, males need to excrete a daily volume of 600–700 mg of uric acid through the glomeruli, and proximal and distal tubules of the kidney; ascending loop; and collecting duct. If kidney functioning is reduced, the probability of hyperuricemia becomes relatively high [33]. Furthermore, in this study, serum creatinine level and hyperuricemia showed a positive association, which is consistent with the existing study results that renal functioning affects serum uric acid levels.

In fact, according to recent studies, an increase in blood lead concentration caused a reduction in estimated glomerular filtration rate (eGFR) even at a blood lead concentration lower than 10.0 μg/dL [34]. In addition to the glomeruli, blood lead causes the atrophy and hyperplasia of the tubular epithelial cells and increases the risk for hyperuricemia by reducing secretory functions to cause interstitial fibrosis and inflammation [7,35,36]. In addition, when blood lead concentration is higher than 1.2 μg/dL, the risk for gout reportedly increases with the reduction in uric acid excretion ability of the kidney [37].

An independent association with hyperuricemia was observed for smoking and γ-GTP level; however, it is too early to assume that both are direct risk factors for hyperuricemia, as existing studies report inconsistent results, suggesting the need to conduct a prospective study [38-40].

The steelmaking company investigated in this study was an independent steelmaker largely divided into steelmaking and rolling processes. The steelmaking process exposes workers to lead fumes during the dissolving process, during which metal containing impurities is melted [41]. In fact, according to a study that measured ambient lead concentration using individual samples during the steelmaking process, most of the samples showed lead concentrations exceeding 50% of the domestic threshold (0.05 mg/m^3^), indicating that the steelmaking process require attention regarding occupational lead exposure, along with the iron making process, in which metal is extracted by dissolving ore [41-43]. In addition, the workers involved in iron-making processes were exposed to mineral dust, noise, hyperthermia, sulfur dioxide (SO_2_), carbon monoxide (CO), and hydrogen sulfide (H_2_S), but none of the causative factors of hyperuricemia identified by previous studies was found.

This study has its significance in providing results that controlled task properties and sex by limiting the subjects to male workers from a steelmaking company known to have the most number of lead handling processes among other metal industries, in comparison with existing studies that did not match task properties while targeting the general public or workers as subjects. It is also significant in that its simultaneous analyses revealed that hyperuricemia is significantly associated with metabolic syndrome and that low levels of blood lead can affect hyperuricemia.

This study has the following limitations: First, it is a cross-sectional study, and a causal relationship between blood lead level, serum uric acid level, and metabolic syndrome could not be established while attempting to explain the intervariable relationships using various studies in the existing literature. Second, recall bias might have existed, as the smoking, drinking, and exercise habits, and drug therapy were examined through surveys; this bias was minimized through the comparison between existing survey data for identical subjects. Third, no survey was conducted on diet related to serum uric acid concentration; it was partially controlled as the workers took the same meal twice a day at the company canteen while working 3 shifts, but a more accurate investigation needs to be conducted. Lastly, as it is an analysis for a particular group, our study was limited in generalizing the results to all population groups. In a group without occupational exposure, identical physiological mechanisms due to blood lead levels were reported [44]. However, a study with a general population group is needed in the future.

At present, with the prevention and management of workplace workers’ cerebrovascular and cardiovascular diseases becoming increasingly important, attention should be given to serum uric acid concentration, which has been regarded as a causal factor only for gout. In particular, from the perspective of occupational environment medicine, hyperuricemia should be managed in individuals who are likely to be exposed to lead over a longer period of time for occupational and environmental reasons. The results of this study suggest that a risk for hyperuricemia exists even at low blood lead concentrations. Thus, it is necessary to conduct a large-scale prospective study to identify any causal relationship.

## Competing interests

The authors declare that they have no competing interests.

## Authors’ contributions

DL and WJC designed the research and performed the statistical analysis. JSO, MKY and SWH collected the data. JWY and SHH revised the manuscript. All of the authors read and approved the final manuscript.
